# Mining of Root-Specific Expression Genes and Their Core Cis-Regulatory Elements in Plants

**DOI:** 10.3390/ijms26041720

**Published:** 2025-02-18

**Authors:** Shan Gao, Yi Chai, Xinyue Zhou, Suhui Chen

**Affiliations:** Shanghai Key Laboratory of Plant Molecular Sciences, College of Life Sciences, Shanghai Normal University, Shanghai 200234, China

**Keywords:** root-specific expression genes, cis-regulatory elements, plants

## Abstract

Mining tissue-specific genes is important for studying the processes of life activities within tissues, and it is a way of finding genes that regulate relevant traits. In recent years, the massive growth of expression data from various tissues has provided important opportunities for the large-scale analysis of tissue-specific genes. We found 489, 276, and 728 RTEGs (root tissue-specific expression genes) using 35 RNA-seq databases in 13 different tissues from three species of plants, e.g., Arabidopsis, rice, and maize, respectively, by bioinformatics methods. A total of 34 RTEGs in rice were found to be conserved in all three species, and 29 genes of them were unreported. Furthermore, 16 newly core cis-acting elements, named REM1-16 (root expression motif), were predicted by four well-known bioinformatics tools, which might determine the root tissue expression pattern. In particular, REM2 is conserved in not only Arabidopsis, but also rice. These cis-acting elements may be an important genetic resource that can be introduced into synthetic memory circuits to precisely regulate the spatiotemporal expression of genes in a user-defined manner.

## 1. Introduction

Roots are vital to plant growth and development as they not only anchor the plant and sense its surroundings, but also anchor and absorb water and nutrients [1]. Two important symbioses in which roots are also involved are the mycorrhizal symbiosis and the root/bacterial mutualism, i.e., the fixation of atmospheric nitrogen. Many tissue-specific genes play key biological roles in organisms and are associated with a wide range of important agronomic traits in crops. However, the systematic identification of conserved RTEGs across species has been largely overlooked. Bridging this research gap holds the potential not only to elucidate core regulatory mechanisms governing root development, but also to identify evolutionarily conserved genetic targets that could be applied to the precise optimization of crop root architecture [2].

To improve the agronomic traits of crops, various types of promoters have been used to induce expression, including constitutive, spatiotemporal, and stress/chemical inducible promoters. Although many studies have successfully used constitutive promoters, they are accompanied by some side effects because the functional properties of the genes themselves are not taken into consideration. For example, the overexpression lines of *OsMADS26* and *OsbHLH142* driven by the *Ubi* promoter have triggered several abnormal phenotypes, including retarded root/shoot growth and sterility [3,4]. These unintended phenotypes underscore a critical need for precision control tools, specifically tissue-specific promoters, which can restrict gene expression in target tissues while maintaining endogenous physiological balance [5].

Emerging opportunities lie in transcriptomic data. RNA-seq has now revolutionized the way the research community studies gene expression. Indeed, this technology offers the possibility of quantifying the expression levels of all genes at once. Significant amounts of RNA-seq data have been released in the past decade. The real challenge shifts from data generation to knowledge extraction. Nevertheless, few studies have systematically mined these resources to identify conserved RTEGs across species [6]. A DNA sequence motif, a subsequence of DNA sequence, is a short (about 6–12 bp), repeated, and conserved pattern of nucleotides, and it has many biological functions such as being a DNA binding site for a regulatory protein and controlling gene expression. There are hundreds of algorithms for motif extraction in the promoters of genes of interest, and these tools can be classified into three classes, such as enumeration, deterministic optimization, and probabilistic optimization [7,8].

We performed a comparative analysis of tissue-specific gene expression patterns through the systematic integration of 35 RNA-seq datasets across three model plant species (*Arabidopsis thaliana*, *Oryza sativa*, and *Zea mays*). Our investigation identified 489, 276, and 728 root-specific expressed genes in Arabidopsis, rice, and maize, respectively, with 34 orthologous RTEGs conserved across all three species, suggesting the evolutionary conservation of these molecular pathways in plant root development. To decipher the transcriptional regulation of RTEGs, we employed four established de novo motif discovery algorithms for a comprehensive analysis of their promoter sequences. This integrative approach identified 16 core cis-regulatory elements, which were named REM1-16. Next, *cis*-regulatory element sequences could be used to fabricate synthetic promoters by either random ligation or rationally designed building block approaches. Synthetic promoters can precisely control the expression of target genes at a specific temporal and spatial distribution [9,10].

## 2. Results

### 2.1. Large-Scale Investigation of Root Tissue-Specific Expressed Genes in Plants

We selected three representative plant models, including one dicotyledonous plant (*Arabidopsis thaliana*) and two monocots (*Oryza sativa*, *Zea mays*), with the aim of identifying RTEGs or even conserved RTEGs to elucidate the presence of conserved pathways controlling root development in plants. To accurately map the expression profiles, we browsed all RNA-seq data in the NCBI database and finally selected 13 tissues covering almost all organs of plants from the vegetative stages to reproductive stages in three species, e.g., root, shoot, leaf, tillering bud, stigma, ovary, pollen, husk, pericarp, shoot apical meristem (SAM), inflorescence meristem (IM), embryo, and endosperm, and we were guided by the principle that the same tissue RNAseq database, in different species, should be chosen to have as similar a developmental period as possible (Supplementary Table S2).

**Figure 1:**
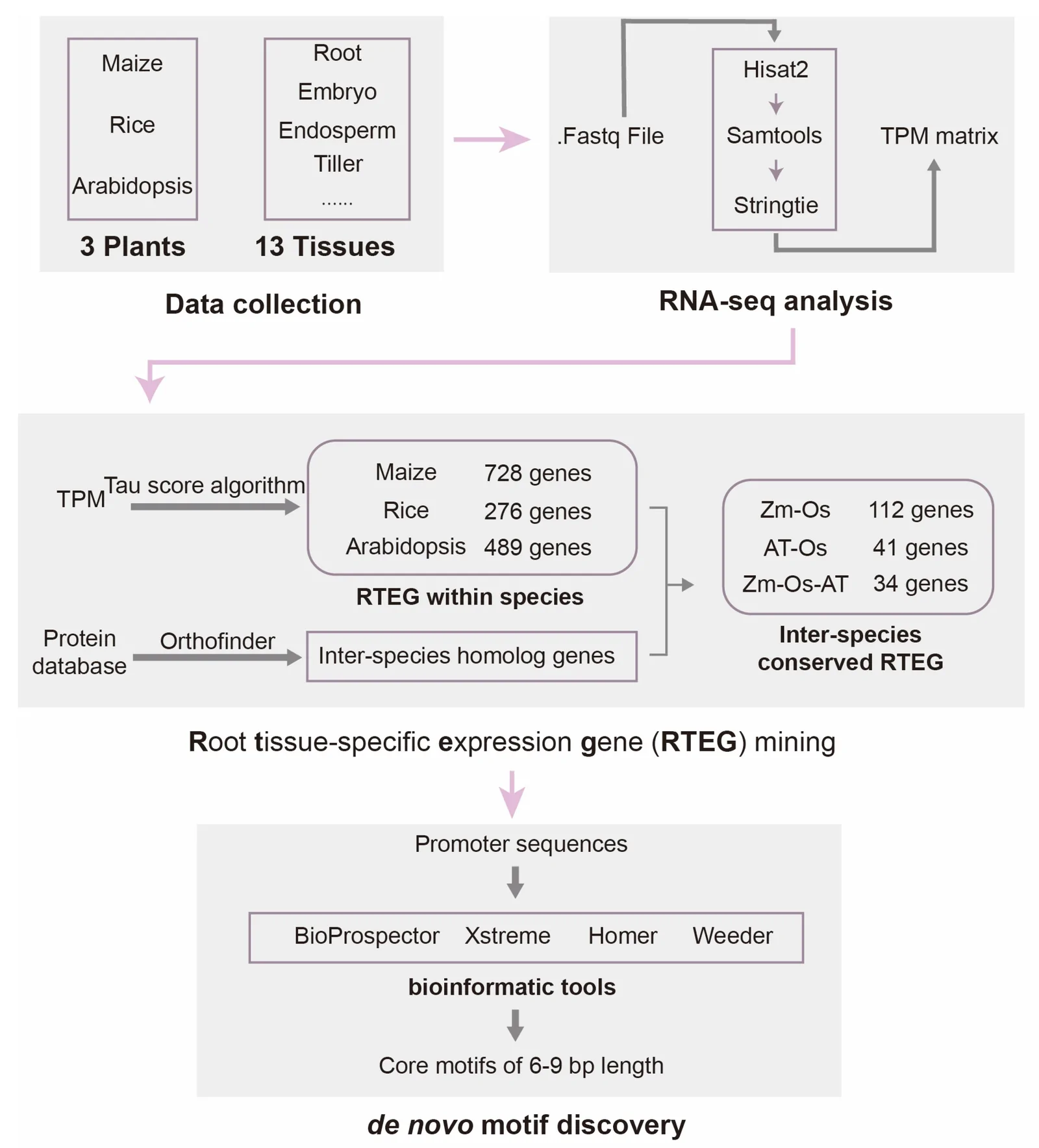
The general scheme for identifying root tissue-specific expression genes and de novo motif discovery.

A total of 35 high-quality RNA-seq data from 29 published papers [11,12,13,14,15,16,17,18,19,20,21,22,23,24,25,26,27,28,29,30,31,32,33,34,35,36,37,38,39] were downloaded, and we conducted an RNA-seq analysis by species (Supplementary Table S1). We performed a Principal Component Analysis (PCA) of RNAseq-based expression data from three plants (Supplementary Figure S1). The results show that all tissues were independent and there was good reproducibility of duplicate biological samples. Furthermore, we isolated RTEGs by improving the tissue specificity index algorithm. The general scheme is shown below (Figure 1).

Bioinformatics analyses included data collection, RNA-seq analysis, root tissue-specific expression gene mining, and de novo motif discovery. Technical details of the work are given in Section 4.

### 2.2. Functional Classification and GO Analysis of RTEGs

In total, 489, 276, and 728 RTEGs were identified from Arabidopsis, rice, and maize, respectively, and were used to perform a gene ontology (GO) analysis (Supplementary Figures S2–S4, Supplementary Table S3). The results show that RTEGs in Arabidopsis are enriched in organic cyclic compound binding, heterocyclic compound binding, catalytic activity, transition metal ion binding, transferase activity, oxidoreductase activity, metal ion binding, hydrolase activity, and cation binding. However, RTEGs in rice are not enriched in organic cyclic compound binding, heterocyclic compound binding, nucleic acid binding, and calcium ion binding. RTEGs in rice are functionally similar to those in maize and are mainly participate in transition metal ion binding, tetrapyrrole binding, substrate-specific transmembrane transporter, oxidoreductase activity, ion binding, heme binding. In short, although RTEG functions are generally similar in different species, there are differences that may arise from the soil in which they live or the specific needs for certain nutrients.

### 2.3. Identification of Conserved RTEGs

**Figure 2:**
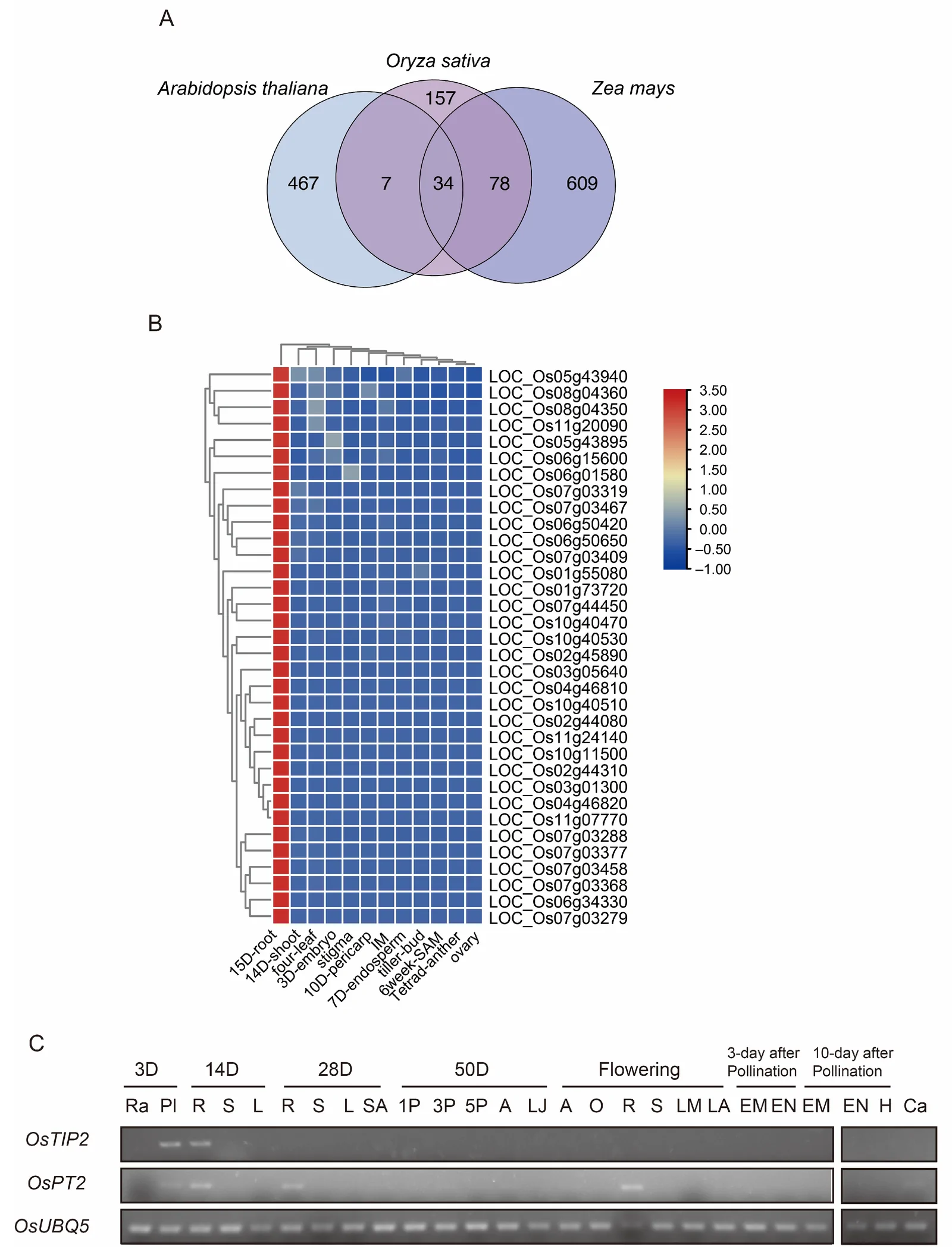
Identification of root tissue-specific expression genes. (**A**) Statistics of number of genes specifically expressed in root tissues of different species and number of conserved homologous genes. (**B**) Expression profiles of conserved root tissue-specific expression genes in rice. Thirteen different tissues from *Oryza sativa* used for profiling are mentioned on bottom of each column, and color bar on right side represents relative expression values. (**C**) Experimental validation of root tissue-specific expression of genes. *OsUBQ5* gene was used as internal control. Twenty-six tissues from different developmental stages were used. Ra: radicle, Pl: plumule, R: root, S: shoot, L: leaf, SA: shoot apical meristem, 1P: < 1 cm inflorescence meristem, 3P: 1–3 cm inflorescence meristem, 5P: 3–5 cm inflorescence meristem, A: anther, LJ: lamina joint, O: ovary, LM and LA: flag leaf at flowering stage (at 10 a.m. and 3 p.m.), EM: embryo, EN: endosperm, H: husk, Ca: callus. Different developmental stages are indicated above black line.

Across species, roots share common functions, such as anchoring the plant, sensing its surroundings, and fixing and absorbing water and nutrients. We inferred all orthogroups in Arabidopsis, rice, and maize using OrthoFinder [40] and isolated conserved RTEGs across species to identify unreported genes that may be involved in the above processes. In the comparison of rice and maize RTEGs, there were 112 conserved RTEGs in rice and 119 conserved RTEGs in maize. In the comparison of rice and Arabidopsis RTEGs, there were 22 conserved RTEGs in Arabidopsis and 41 conserved RTEGs in rice. Surprisingly, we identified 34 conserved RTEGs in rice, whose homologs in Arabidopsis and maize also exhibit root-specific expression patterns (Figure 2A,B). This evolutionary conservation strongly suggests that these genes may function in conserved regulatory pathways in roots. Notably, 29 of the 34 RTEGs have not been previously reported, highlighting their potential as novel candidates for future functional studies. Moreover, we collected 26 different tissues at various development stages to perform RT-PCR to validate the accuracy of the bioinformatic analyses (Figure 2C). The results show that *OsTIP2* was expressed in roots only at an early stage, whereas *OsPT2* was expressed in roots in different stages of plant life.

### 2.4. Motif Discovery

Based on the RTEGs obtained from rice, maize, and Arabidopsis, we extracted a total of 1493 gene promoters from these three species and attempted to uncover the key motifs that regulate the spatial expression of the genes, particularly those in the root tissues, using bioinformatics tools. We selected four representative software packages for de novo cis-acting element analysis, namely BioProspect [41], Weeder [42], Homer [43], and Xstreme [44], and inferred a total of 523 root-specific cis-acting elements. Of these, 40 cis-acting elements each for RTEGs in Arabidopsis, maize, and rice were obtained using a BioProspector analysis. The numbers of cis-acting elements of RTEGs in Arabidopsis, maize, and rice obtained by Xstreme analysis were 36, 28, and 27, respectively. The numbers of cis-acting elements of RTEGs obtained by Homer analysis were 79, 89, and 81, respectively. The numbers of cis-acting elements of RTEGs obtained by Weeder analysis were 24, 21, and 18, respectively. Among them, the BioProspector, Xstreme, and Homer analyses were carried out based on a comparison between RTEGs and embryo-specific expression genes or endosperm-specific expression genes as the background, whereas the Weeder analysis directly mined cis-acting elements for RTEGs.

Since the lengths of sequences obtained from different analysis software vary, we performed sequence continuity truncation of cis-acting elements obtained from software analysis, with 6–9 bp as the main length, in an attempt to find sequences that appeared in more than one software. Through Weeder, XSTREME, Homer, and BioProspector, we identified a total of 91 cis-acting elements, named REM1-91. Sixteen of these key sequences are shown in Table 1, of which Arabidopsis has seven key motifs, named REM1-7; maize has six key motifs, REM8-13; and rice has four root-specific expression cis-acting elements, REM2 and REM14-16. One key motif, REM2, exists in both Arabidopsis and rice. The results show that most of the key sequences exhibited species specificity, and only very few sequences were conserved among species, implying that gene expression patterns in plants may be determined by different sequences and mechanisms.

**Figure 3:**
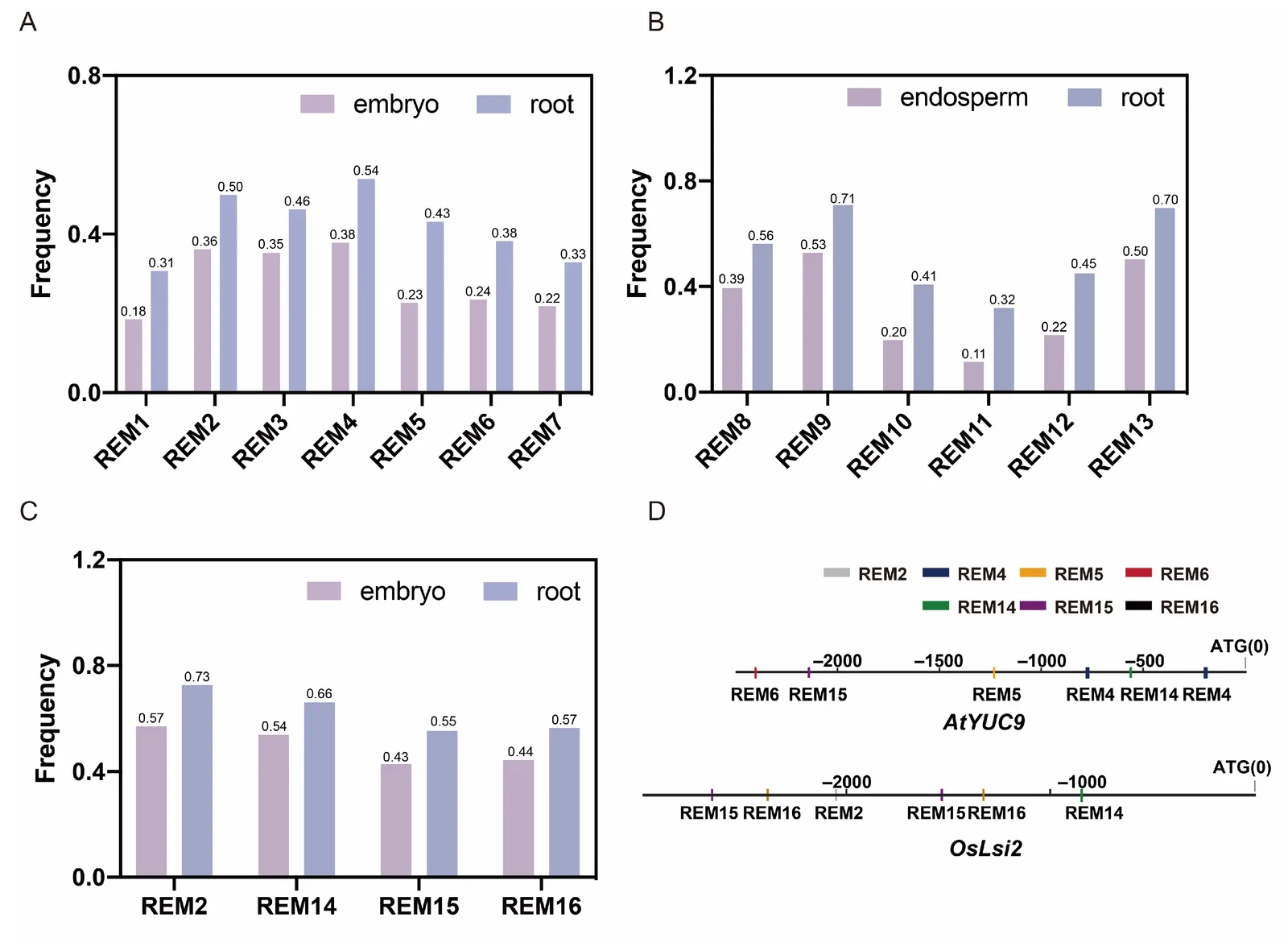
Frequency and positional distribution of 16 core motifs at promoters of tissue-specific expressed genes. Comparison of REM frequencies on promoters of different tissue-specific expressed genes in Arabidopsis (**A**), maize (**B**), and rice (**C**). (**D**) Distribution of REM positions in promoters of two root tissue-specific expression genes.

In addition, we analyzed 16 key motifs, namely REM1-REM16, in the three species to compare their frequency differences in embryo/endosperm and root-specific expressed gene promoters. This was calculated as follows: Frequency of occurrence = number of tissue-specific expressed genes containing the key motif in their promoter sequence/total number of tissue-specific expressed genes × 100%. Through the analysis, we found that the key motifs were distributed in the promoters of embryo/endosperm-specific expressed genes in addition to the RTEGs, but the frequency of occurrence in the RTEGs was significantly higher than that in the other tissues (Figure 3A–C). It is implied that REM1-16 may be the key motifs determining the root expression of the genes.

To further clarify the functions of REM1-16, we analyzed the promoters of some reported RTEGs. We found that two REM4 motifs, one REM5, 6, 14, and 15 were present in the promoter sequence of *AtYUC9* (AT1G0418), a reported Arabidopsis RTEG that promotes the synthesis of ethylene and auxin. On the other hand, *OsLsi2* (LOC_Os03g01700) is a rice RTEG encoding the silica transport protein. The predicted core motifs REM2 and REM14-16 all appeared in the promoter sequence of *OsLsi2*. The results indicate that REM1-16 may be the core cis-acting elements determining the spatial expression of the gene in the roots.

## 3. Discussion

In this study, 35 RNA-seq databases from 13 tissues in three species were used to identify the RTEGs on a large scale using the improved tissue specificity index (τ). We found 489, 276, and 728 root RTEGs in Arabidopsis, rice, and maize, respectively, of which 34 RTEGs in rice were found to be conserved in all three species. Furthermore, 16 newly reported core cis-acting elements, named REM1-16, were predicted by four well-known bioinformatics tools from the RTEGs of three plants, which may be useful in determining the root tissue expression patterns genes as their frequency of occurrence in the promoter sequence of RTEGs was much higher than that of other tissue-specific expressed genes. Importantly, REM2 is conserved not only in Arabidopsis, but also in rice. In future studies, the 16 core cis-acting elements deserve further experimental validation.

The conserved expression profiles of 34 evolutionarily conserved RTEGs suggest their potential involvement in fundamental regulatory pathways governing plant root development or signaling transduction. Notably, 29 of these 34 RTEGs have never been unreported before and are therefore priority targets for mechanistic studies on root-specific biological processes.

Although constitutive promoters have been widely adopted in plant biotechnology for strong and persistent transgene expression, their indiscriminate activity across all tissues and developmental stages often leads to unintended pleiotropic effects, such as impaired plant growth or metabolic burden. In contrast, gene regulatory systems employ cis-acting elements (e.g., conserved transcription factor-binding motifs) enable the precise spatiotemporal control of gene expression by integrating environmental or developmental signals. However, engineering such complexity in transgenic systems remains challenging. Synthetic gene circuits—modular assemblies of genetic components designed to process input signals and regulate output genes in a user-defined manner—offer a promising solution. By incorporating root-specific cis-acting motifs (identified in this study) as core regulatory elements, these circuits could dynamically fine-tune gene expression in a tissue-dependent manner, mimicking natural precision while avoiding the limitations of constitutive overexpression [45].

## 4. Materials and Methods

### 4.1. Plant Materials

Rice Zhonghua11 (ZH11, *Oryza sativa japonica*) plants were grown in Shanghai under standard paddy conditions.

To identify the expression pattern, 26 tissues from different developmental stages were collected, including the plumule and radicle (3 days after germination), root, shoot, and leaf (14 days, 28 days, and flowering stage), shoot apical meristem (SAM, 28-day-old plant), inflorescence meristem (less than 1 cm, 1–3 cm, and 3–5 cm), unmature pollen, lamina joint, anther, ovary, flag leaf at the flowering stage (at 10 a.m. and 3 p.m.), embryo (3 and 10 days after fertilization), husk (10 days after fertilization) and endosperm (3 and 10 days after fertilization), and callus.

### 4.2. Data Collection and RNA-Seq Analysis

A total of 35 pieces of high-quality RNA-seq data from 3 species of plants, including one dicotyledonous plant (*Arabidopsis thaliana*) and two monocots (*Oryza sativa*, *Zea mays*), were collected from 29 published papers [11,12,13,14,15,16,17,18,19,20,21,22,23,24,25,26,27,28,29,30,31,32,33,34,35,36,37,38,39]. In Supplementary Table S1, detailed information, e.g., species, tissue name, bioproject, sequence read archive (SRA), and publication, is listed.

RNA sequencing data were mapped to the reference genome (IRGSP-1.0 for rice, AGPv4 for maize, TAIR10 for Arabidopsis) using HISAT2, and StringTie was used to calculate the transcripts per million (TPM) values [46]. OrthoFinder was used to group genes into orthogroups [40].

TPM-matrix was further used as input for Principal Component Analysis (PCA), heatmap, and Gene Ontology (GO) enrichment analysis. FactoMineR, factoextra, and ggrepel (R package) were used to perform PCA and visualization [47]. We used TBtools to draw a heatmap [48]. In 15 November 2024, the agriGO website (http://systemsbiology.cau.edu.cn/agriGOv2/index.php) was used to perform GO enrichment analysis to identify enriched cellular components, molecular function, and biological process categories [49].

### 4.3. Tissue-Specific Expression Gene Analysis

The tissue specificity index (τ) [50] is defined as follows:

τ=∑i=1N(1−xi^)N−1xi^=log10((xi+1)/log10(xmax+1)

*N* is the number of tissues, and xi^ is calculated based on the expression value in different tissues i ($x_{i}$) and the maximal expression value ($x_{max}$). The tau score ranges from 0 to 1. To eliminate the effect of $x_{max}$ and $x_{sec}$ on the tissue specificity index (τ) under extreme conditions, tissue-specific expression gene screening is carried out based on the following criteria, where τ needs to be greater than 0.85 in all cases.

If the value of the second high expression ($x_{sec}$) is not equal to 0, ${2<x}_{max}\leq 20$, and $x_{max}/x_{sec}\geq 4$;

If the value of the second high expression ($x_{sec}$) is not equal to 0, ${20<x}_{max}$, and $x_{max}/x_{sec}\geq 10$;

If $x_{sec}$ = 0, $x_{max}\geq 1$.

### 4.4. RNA Extract and RT-PCR

Total RNAs of different tissues mentioned above were extracted by Trizol reagent (Invitrogen, Carlsbad, USA) and used to synthesize cDNA through reverse transcription (Takara, Kusatsu, Japan). RT-PCR was conducted in a total volume of 20 μL containing 10 μL Taq mix, 6 μL cDNA (diluted 30 times), 1.5 μL primers (10 mM), and 2.5 μL double-distilled water. *UBQ5* gene was used as an internal control (primers: ACCACTTCGACCGCCACTACT, ACGCCTAAGCCTGCTGGTT), and transcription levels of *OsPT2* and *OsTIP2* were examined using primers *OsPT2*-F, R (GGTATGCTCATGACGCTGCT, TTGGGCGATCGCTTCTTGG), and *OsTIP2*-F, R (GGAAGCTTGGGTGACTCCTT, AAACGCCCACGAACAGGG). All examinations were conducted with three biological and technological replicates.

### 4.5. De Novo Motif Discovery

Promoter sequences of RTEGs were first extracted from maize, rice, and Arabidopsis. Among them, the 3 kb sequence of RTEGs before ATG was extracted from maize and rice, and the 2.5 kb sequence of RTEGs before ATG was extracted from Arabidopsis. Xstreme v5.4.1 [44], Weeder 2.0 [42], BioProspector [41], and Homer v4.11 [43] were used in this study. Each bioinformatic tool was used to search for both strands with default parameters. The promoter sequence of either embryo tissue-specific expression gene or endosperm tissue-specific expression gene was used as background sequence.

## Figures and Tables

**Table 1:** Sixteen core motifs within the promoter sequences of root tissue-specific expression genes.

Motif Name	Motif Sequences	Motif Length	Species	Xstreme	Homer	Bioprospector	Weeder
REM1	AAAATTTGA	9	Arabidopsis	**	**	NA	NA
REM2	AATTAATC	8	Arabidopsis	NA	*	NA	NA
			Rice	NA	**	*	NA
REM3	AATTACG	7	Arabidopsis	**	*	*	NA
REM4	ACATGCA	7	Arabidopsis	NA	**	**	Y
REM5	ACGTGC	6	Arabidopsis	NA	**	NA	NA
REM6	AGCACG	6	Arabidopsis	*	**	NA	Y
REM7	CGACCG	6	Arabidopsis	*	*	NA	NA
REM8	AATCGAT	7	Maize	NA	**	*	NA
REM9	AGCTAGC	7	Maize	*	*	**	NA
REM10	AGCTAGCT	8	Maize	*	*	**	NA
REM11	AGCTAGCTA	9	Maize	*	*	**	NA
REM12	ATCGATCG	8	Maize	*	*	**	NA
REM13	ATCGATC	7	Maize	*	*	**	NA
REM14	ATACATA	7	Rice	NA	NA	*	NA
REM15	ATACATAT	8	Rice	*	**	**	NA
REM16	ATTAATTC	8	Rice	NA	**	**	NA

Four well-known bioinformatics tools, including Xstreme, Homer, BioProspector, and Weeder, were used for de novo motif discovery. * indicates that the sequence occurs either once in root embryo or root endosperm, ** indicates that the sequence occurs in both root embryo and root endosperm, and NA indicates that it does not occur in either case. ”Y” is the motif identified by Weeder.

## Data Availability

The original contributions presented in this study are included in the Supplementary Material. Further inquiries can be directed to the corresponding author.

## Supplementary Materials

The supporting information can be downloaded at https://www.mdpi.com/article/10.3390/ijms26041720/s1.

## Abbreviations

The following abbreviations are used in this manuscript:

RTEGs	Root tissue-specific expression genes
REM	Root expression motif
