# Supplementary material for: Mining of Root-Specific Expression Genes and Their Core Cis-Regulatory Elements in Plants
Source: Int J Mol Sci. 2025 Feb 18;26(4):1720. doi: 10.3390/ijms26041720 (PMC11855845; doi:10.3390/ijms26041720)
Supplement: Supplementary file 1 [file ijms-26-01720-s001.zip › Supplementary Figure.pdf]

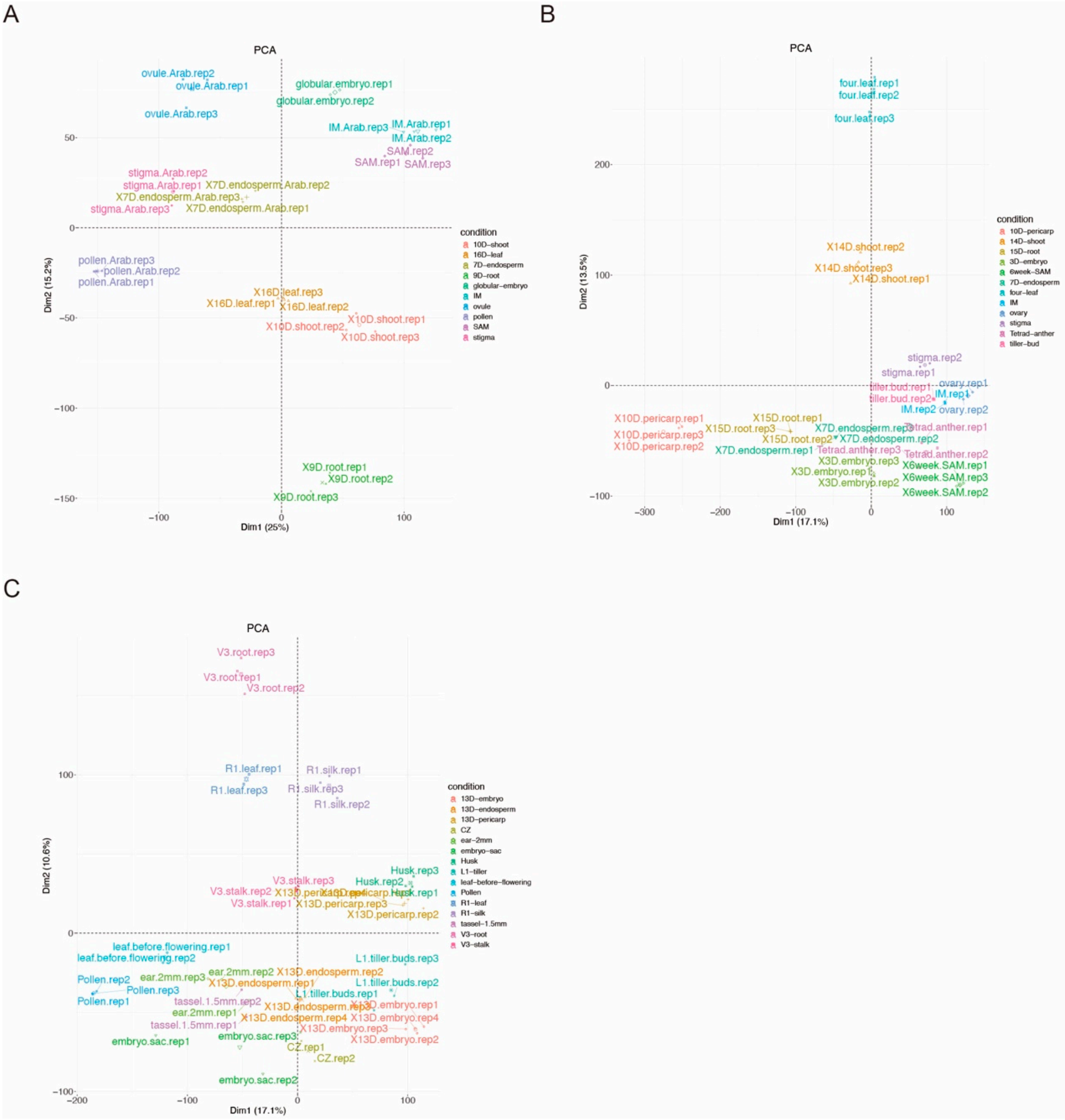

Supplementary Figure 1. Principal Component Analysis (PCA) of RNAseq-based expression data from three plants.

PCA plots of RNA-seq data show the characteristics of samples according to TPM (Transcripts per million) levels in Arabidopsis (A), rice (B), and maize (C), and each dot indicates a sample.

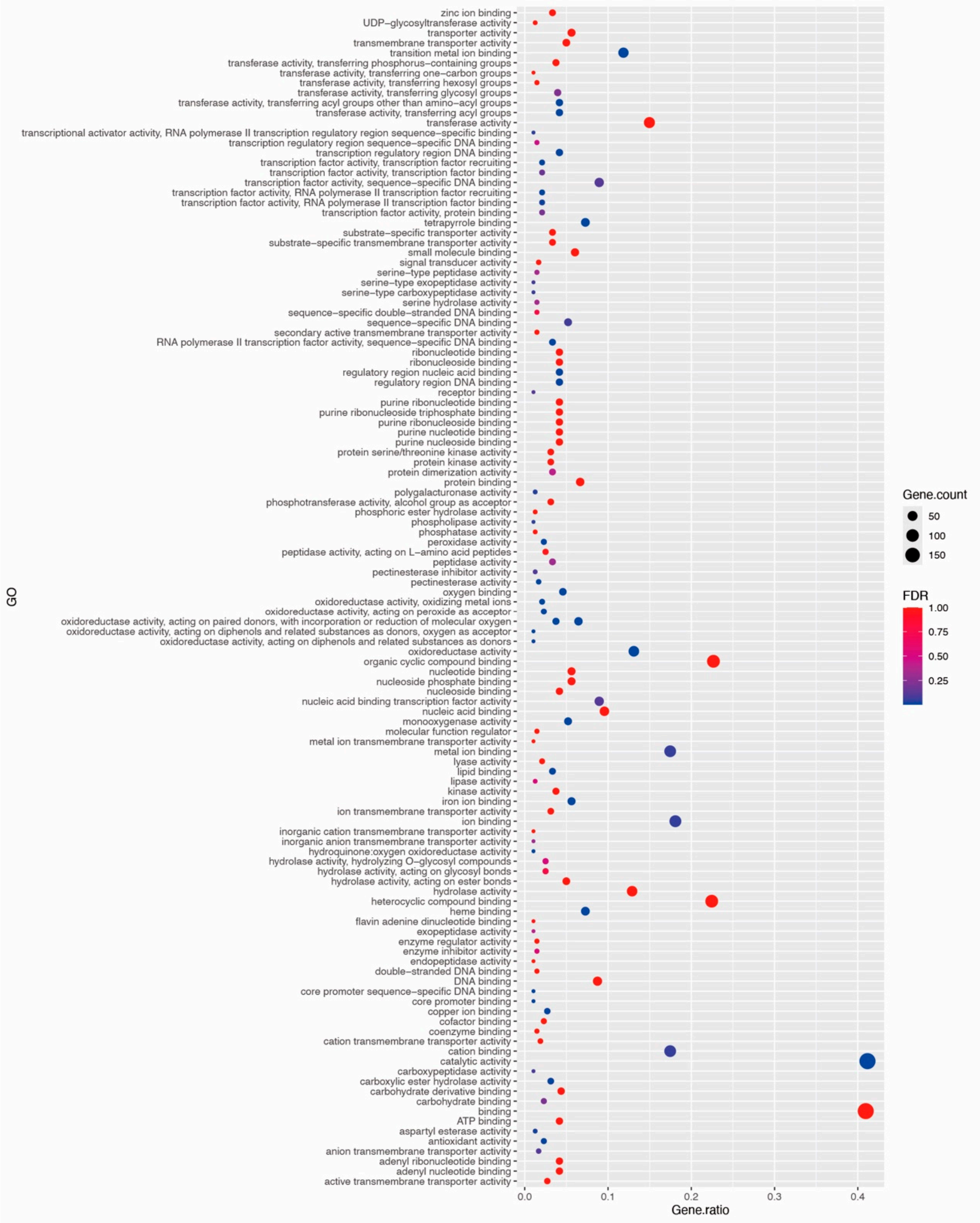

Supplementary Figure 2. GO enrichment analysis of RTEGs in Arabidopsis.

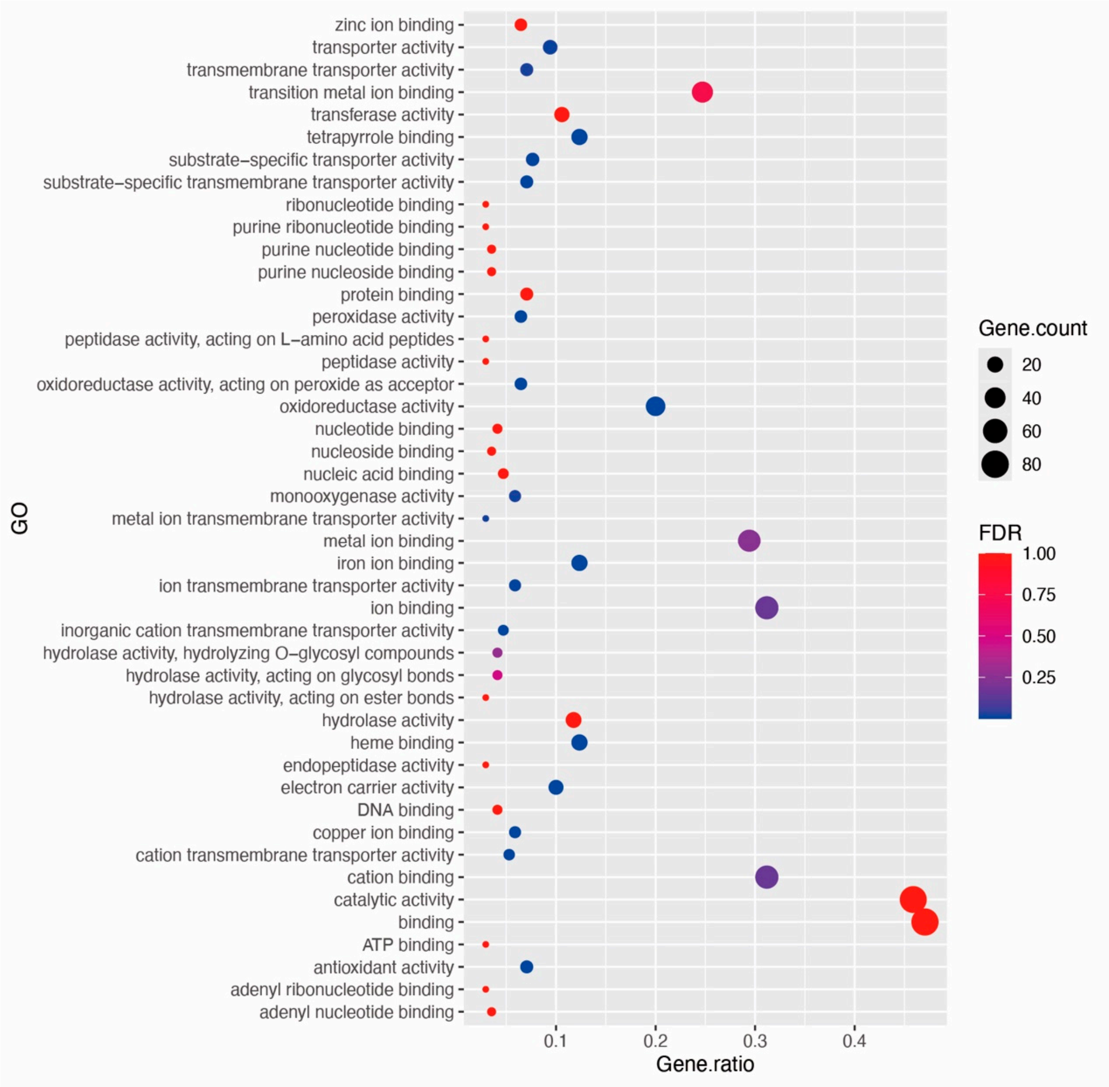

Supplementary Figure 3. GO enrichment analysis of RTEGs in rice.

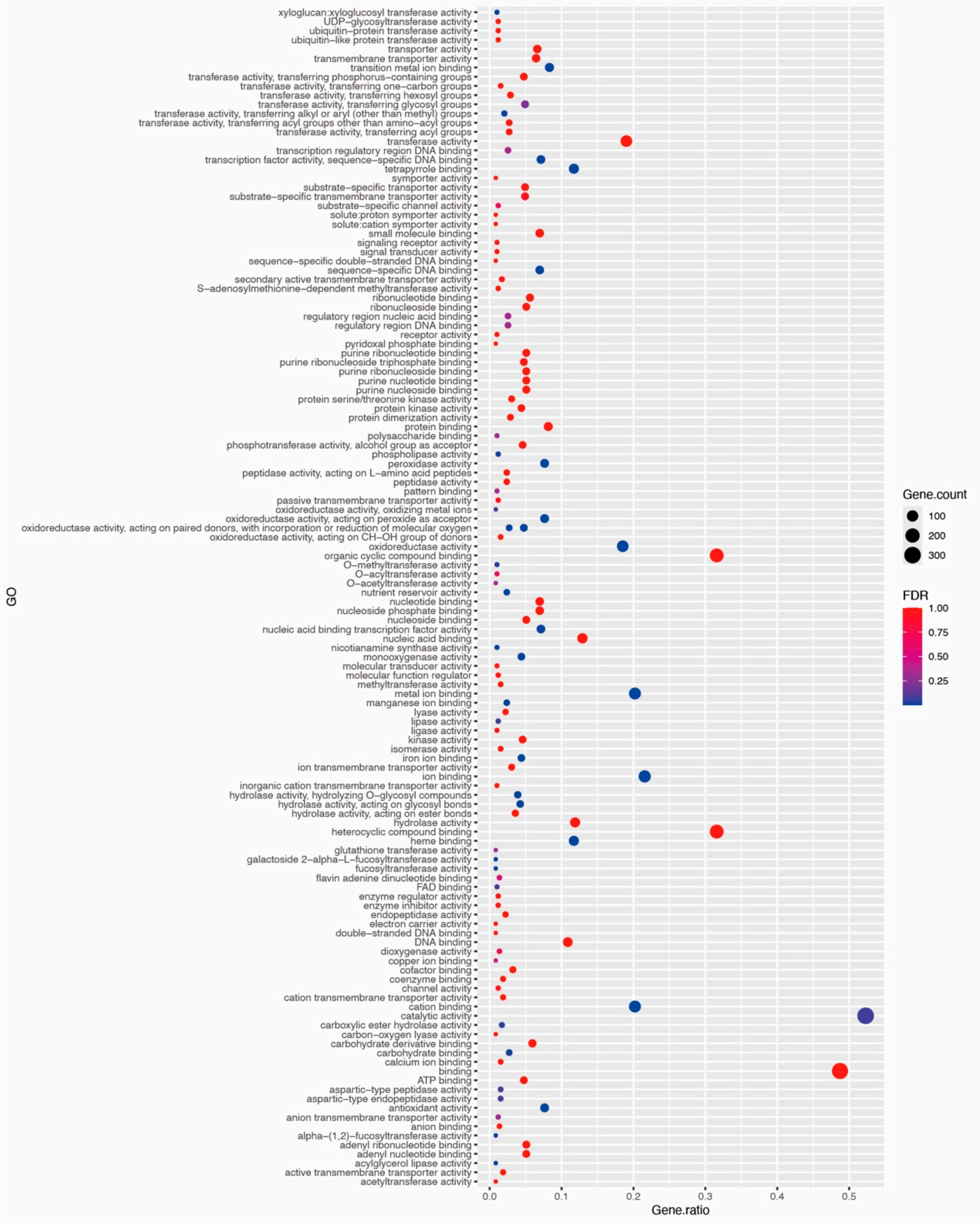

Supplementary Figure 4. GO enrichment analysis of RTEGs in maize.
